# Predator-induced morphological traits determine escape performance in a larval amphibian species

**DOI:** 10.1098/rsos.260411

**Published:** 2026-10-14

**Authors:** Naru Matsuura, Raj R. Malinda, Shin Fukuda, Hiroaki Kawashima, Osamu Kishida

**Affiliations:** ^1^ Graduate School of Environmental Sciences, Hokkaido University, Sapporo, Japan; ^2^ Graduate School of Engineering, Hokkaido University, Sapporo, Japan; ^3^ Graduate School of Information Science, University of Hyogo, Kobe, Japan; ^4^ Wakayama Experimental Forest, Field Science Center for Northern Biosphere, Hokkaido University, Wakayama, Japan

**Keywords:** predator-induced morphology, inducible defence, escape speed, swimming efficiency, anuran tadpole

## Abstract

Although interspecific morphological diversity in aquatic animals often reflects swimming-related functional trade-offs, whether intraspecific morphological variation, often generated by phenotypic plasticity, also reflects similar trade-offs remains unclear. We tested this hypothesis using *Rana pirica* tadpoles, which exhibit a dragonfly-induced phenotype with a deeper tail and a salamander-induced phenotype with both a deeper tail and a swollen body. Fast-start swimming speed was highest in the dragonfly-induced phenotype, intermediate in the salamander-induced phenotype and lowest in the non-induced phenotype. Because swimming speed is influenced by both morphology and body-motion effort, we quantified motion–speed efficiency (*E_ms_*) as an index of morphological effects on swimming performance. Both predator-induced phenotypes exhibited higher *E_ms_* than the non-induced phenotype, despite similar tail-motion quantity among phenotypes, with the highest value in the dragonfly-induced phenotype. These results indicate that the shared deeper tail more efficiently converts body-motion effort into swimming speed, whereas in the salamander-induced phenotype, this function is partly offset by increased hydrodynamic drag caused by its swollen body, a morphology that protects against swallowing by gape-limited salamander larvae. These results demonstrate that predator-induced morphologies involve functional trade-offs between swimming escape and physical protection and suggest that such trade-offs contribute to morphological diversification through phenotypic plasticity.

## 1. Introduction

The interspecific morphological diversity of organisms represents the outcome of evolutionary diversification that is shaped by ecological trade-offs [1–3]. In aquatic animals, particularly among species that rely on swimming as their primary mode of locomotion, trade-offs involving swimming performance are especially widespread and evolutionarily significant [4]. Swimming is fundamental to survival for nektonic and actively swimming aquatic animals—it determines how effectively they escape predators, capture prey and move through their environments [5–6]. However, morphological traits that enhance swimming performance often compromise other functions, such as protection or stability, owing to biomechanical constraints inherent in body design [7–9].

For instance, fish species with streamlined bodies or large propulsive structures exhibit strong thrust and experience reduced hydrodynamic resistance, which are advantageous for predator evasion and prey capture [5,9–10], but their lack of external protective structures makes them vulnerable to direct attacks or reduces manoeuvrability [10]. Conversely, fish species possessing defensive morphologies—such as spines or rounded bodies—gain advantages in protection but suffer from increased hydrodynamic resistance or reduced thrust during swimming [11–13]. Thus, the interspecific morphological diversity observed among mobile aquatic animals often reflects evolutionary compromises that balance swimming performance against other ecological functions.

While interspecific diversity reflects long-term evolutionary diversification, morphological variation also occurs within species, arising from both genetic differences among individuals and phenotypic plasticity within individuals [14–15]. In particular, morphological changes induced plastically by environmental conditions can be drastic, and such plasticity constitutes a major source of remarkable intraspecific morphological diversity in aquatic animals [16–18]. Some plastic morphological changes are accompanied by corresponding changes in swimming performance [19]. This suggests that, just as interspecific morphological diversity has evolved under trade-offs between swimming performance and other functions, morphological variation arising from phenotypic plasticity is also shaped by similar functional constraints. However, few empirical studies have tested whether such plastic variation in morphology involves performance trade-offs. Such tests are crucial for understanding how performance-based trade-offs operate as a fundamental mechanism underlying morphological diversification across multiple levels of biological organization—from variation among species to variation within species.

To address this research gap, we focused on predator-induced morphological plasticity, one of the most widely studied and ecologically relevant forms of phenotypic plasticity [20]. Our working hypothesis is that predator-induced morphologies have evolved under trade-offs between swimming performance and other functions. When fast-start swimming provides an effective means of escaping from predators, prey are expected to express morphologies that enhance fast-start performance under predation risk, even at the cost of reduced defensive protection (i.e. the ability to resist direct attacks). Conversely, when protection serves as a more effective survival strategy than escape, prey are expected to express morphologies that enhance defensive functions under predation risk, even at the expense of swimming performance. By testing this hypothesis using inducible defensive morphologies in anuran tadpoles, a well-established model system for studying predator-induced plasticity, we aimed to investigate how performance-based trade-offs contribute to the formation of adaptive morphological variation.

Several studies on inducible defences in anuran tadpoles have shown that individuals expressing predator-induced morphological traits exhibit greater fast-start swimming speed than conspecifics lacking these traits (e.g. [21–23]), suggesting that such traits enhance fast-start swimming performance [21,23–27]. However, the role of morphology in fast-start swimming remains elusive. One major reason is that potential behavioural effects (i.e. effects of body-motion effort) have rarely been disentangled from morphological effects. Individuals that grow up in predator-present conditions (hereafter, predator-induced phenotypes) not only develop morphological defences, but also exhibit behavioural defences (e.g. behavioural adjustments in body motion during escape responses [28]). If, upon encountering a predator, they move their propulsive structure (e.g. tail fins in amphibian larvae) more forcefully or frequently compared to individuals grown in predator-free conditions (hereafter, non-induced phenotypes), this behavioural difference alone could result in greater fast-start swimming speed (discussed in [19,21]). Therefore, to evaluate whether changes in morphology per se lead to changes in fast-start swimming speed, it is necessary to analyse swimming performance while accounting for the magnitude of motion of the propulsive structure. Demonstrating that predator-induced phenotypes convert propulsive effort into swimming speed more effectively than non-induced phenotypes would strongly support the hypothesis that predator-induced morphological traits enhance fast-start performance.

Therefore, to test this prediction in a well-established model system of predator-induced morphological plasticity, we examined tadpoles of *Rana pirica*, which exhibit two distinct predator-induced phenotypes. The dragonfly-induced phenotype develops a deeper tail that is hypothesized to enhance fast-start swimming performance (predator: *Aeshnid* dragonfly naiads). The salamander-induced phenotype develops both a deeper tail and a swollen body that likely enhance protective rather than locomotor functions—the swollen body preventing swallowing by gape-limited salamander larvae, *Hynobius retardatus* (figure 1) [29]. Tail enlargement, a trait shared by both predator-induced phenotypes, represents a typical predator-induced morphological trait widely observed in other anuran species [30–33].

**Figure 1.**
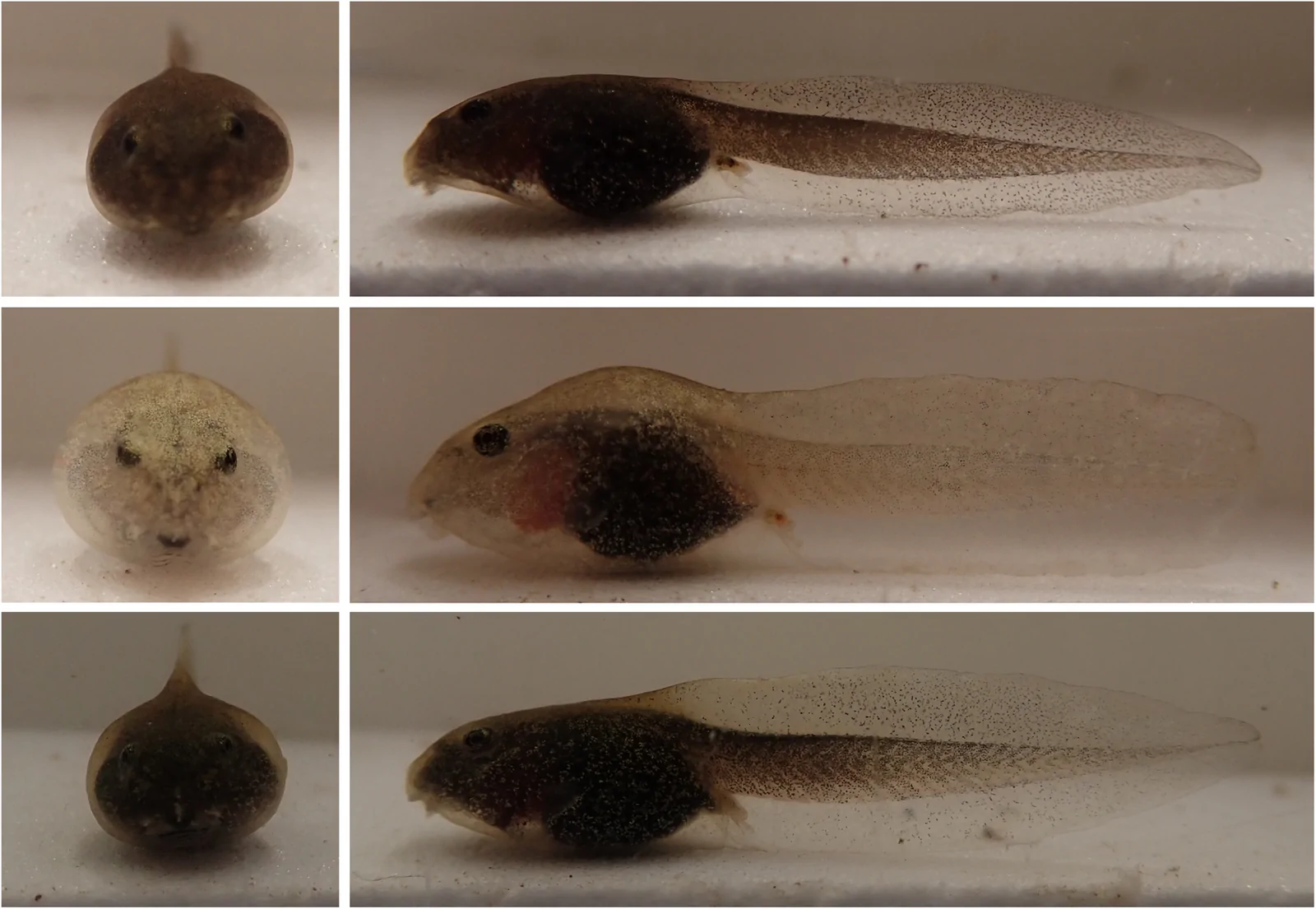
Photographs of non-induced and predator-induced phenotypes in *R. pirica* tadpoles. Left column: front view; right column: lateral view. Upper: non-induced phenotype; middle: salamander-induced phenotype characterized by a deeper tail and larger body; bottom: dragonfly-induced phenotype characterized by a deeper tail.

We predicted that the deeper tails of both predator-induced phenotypes would enhance the effectiveness with which body-motion effort is converted into swimming speed and thereby enhance fast-start performance relative to the non-induced phenotype, whereas the swollen body of the salamander-induced phenotype would increase hydrodynamic drag and reduce this efficiency. To isolate the morphological effect on swimming speed from behavioural variation, we quantified motion–speed efficiency (*E_ms_*)—defined as the effectiveness with which body-motion effort is converted into swimming speed—and compared it among the three phenotypes.

## 2. Materials and methods

The study consisted of two phases. First, we obtained three phenotypes of *Rana pirica* tadpoles by rearing individuals in the presence of salamander larvae (*Hynobius retardatus*), dragonfly naiads (*Aeshna crenata*) or under predator-free conditions [34]. Second, we conducted an escape experiment to evaluate the speed and motion of fast-start swimming in all three phenotypes.

### 2.1. Collection and maintenance

Eggs of *R. pirica*, *H. retardatus* and *A. crenata* naiads were collected from ponds in Hokkaido, Japan, in May 2023. Two pre-hatching *R. pirica* egg masses were collected from a pond in Uryu Experimental Forest, Hokkaido (44°44.38 N,142°19.55 E) on 15 May. Across Hokkaido Island, *R. pirica*, *H. retardatus* and large *Aeshnid* dragonflies are all widely distributed and frequently co-occur in lentic breeding habitats. Although predator composition and density vary among individual ponds, *R. pirica* tadpoles throughout Hokkaido are commonly exposed to both *H. retardatus* and *A. crenata* naiads. We therefore consider the focal population used in this study to represent a population with an evolutionary history of interaction with both predator types. Five *H. retardatus* egg sacs and 10 *A. crenata* naiads were collected from ponds in Teshio Experimental Forest, Hokkaido (45°04.19 N, 142°01.26 E) on 23 May. Collected eggs of *R. pirica* and *H. retardatus* were each maintained in a separate stock tank (33.4 cm × 20 cm × 10 cm high) filled with 2 l of aged water. *Aeshna crenata* naiads were kept individually in a 200-ml polypropylene cup filled with 100 ml of aged water to prevent cannibalism. *Rana pirica* eggs were maintained in a 16°C laboratory room, while *H. retardatus* eggs were kept at 5°C in refrigerators. The difference in keeping temperatures was intended to replicate the natural phenology of predation between *R. pirica* tadpoles and *H. retardatus* larvae, while also accommodating experimental scheduling constraints. *Rana pirica* tadpoles hatched on 16 May 2023 and were fed rabbit chow ad libitum every 3 days until the start of the induction experiment. *Hynobius retardatus* larvae hatched on 5 July 2023, shortly before the induction experiment. *Aeshna crenata* naiads were fed small-sized *R. pirica* tadpoles and bloodworm ad libitum every third day. Water in all tanks was changed every third day. All stock tanks were placed in a natural light–dark environment.

### 2.2. Induction protocol

In our study, three phenotypes of *R. pirica* tadpoles—non-induced, salamander-induced and dragonfly-induced—were obtained through a 12-day laboratory experiment conducted between 6 and 17 July, 2023. The experimental units were 13-L tanks (36 cm × 24 cm × 14 cm high) filled with 4 l of aged water that contained one saucer-shaped colander (15 and 8 cm in diameter at the top and bottom, respectively). On 6 July, we randomly assigned 30 tadpoles to each tank and placed them outside the colander. The mean (± s.d.) body length of a subsample measured prior to the experiment was 8.43 ± 1.44 mm (*n* = 60). The non-induced and dragonfly-induced treatments were each replicated in three tanks, whereas the salamander-induced treatment was replicated in five tanks. An additional replication in the salamander-induced treatment was necessary because salamander larvae frequently bite or prey upon tadpoles, resulting in tail damage and reducing the number of individuals with intact tails available for an escape experiment. To obtain salamander-induced and dragonfly-induced phenotypes, tadpoles were reared with either *H. retardatus* salamander larvae or *A. crenata* dragonfly naiads, respectively. The non-induced phenotypes were obtained by rearing them without predators. Expression of dragonfly-induced phenotypes in *R. pirica* tadpoles requires only a remote cue (chemical cues) from dragonfly naiads, whereas expression of the salamander-induced phenotypes requires a cue from salamander larvae in close proximity (e.g. tactile and close chemical cues) to *R. pirica* tadpoles [34]. For dragonfly-induced treatment, one dragonfly larva (mean ± s.d., total length = 45.0 ± 6.76 mm, *n* = 3) was introduced inside the colander, which segregated it from the tadpoles, so that predation was prevented. To standardize predator cue levels among tanks, dragonfly larvae were used only if they had recently consumed tadpoles. Individuals that failed to feed either before or during the experiment were replaced with fed larvae. For salamander-induced treatment, however, 15 newly hatched salamanders (hatched on 5 July, mean ± s.d., total length = 18.9 ± 1.56 mm, *n* = 30) were introduced into the tank outside the colander, that is, into the same space as the tadpoles. During the induction experiment, *R. pirica* tadpoles in all treatments were fed rabbit chow and bloodworms every other day. In the salamander-induced treatment, salamander larvae were fed bloodworms or tadpoles every other day. In the dragonfly-induced treatment, two small *R. pirica* tadpoles were placed in the colander every other day as prey for *A. crenata* naiads. The induction experiment was conducted in a 16 °C laboratory room under natural light–dark conditions.

### 2.3. Escape experimental protocol

To examine the escape responses of the three phenotypes of tadpoles, we conducted escape trials on 17 July, 2023. The experimental units were a white plastic tub (45 cm × 33 cm × 7 cm height) filled with 4 l of aged water, to maintain a 2.0 cm water depth. For each treatment, 10 tadpoles with intact tails and similar body length were selected, with 1–4 individuals sampled from each tank. All selected individuals were at the developmental stage 25 [35], at which limb buds have not yet developed, and thus no protrusions are present that could affect swimming performance. Then, one tadpole was introduced into the experimental tank at a time. After a 1-minute acclimation period, escape responses were elicited by rapidly moving a magnet towards the tadpole from a distance of 1–5 cm, approaching from the lateral or posterior direction to simulate a predator's attack (supplementary material S1). Each tadpole underwent five escape trials, with a 3-minute interval between trials to ensure sufficient recovery before each attempt. A magnet was moved by attaching another magnet beneath the experimental tank and manipulating it manually from outside the tank (figure 2*a*). To ensure clear visibility during video analysis, a blue magnet with a radius of 4.0 cm was used to distinguish it from the black-coloured tadpoles. Escape responses of tadpoles were captured by video cameras (Panasonic HC-VX992M) from above, with the frame rate at 60 frames per second (fps) and a resolution of 1920 × 1080 pixels. To account for potential variation in escape-trial conditions that could influence swimming performance, we also quantified stimulus speed and the distance between each tadpole and the nearest tank wall at the onset of escape.

**Figure 2.**
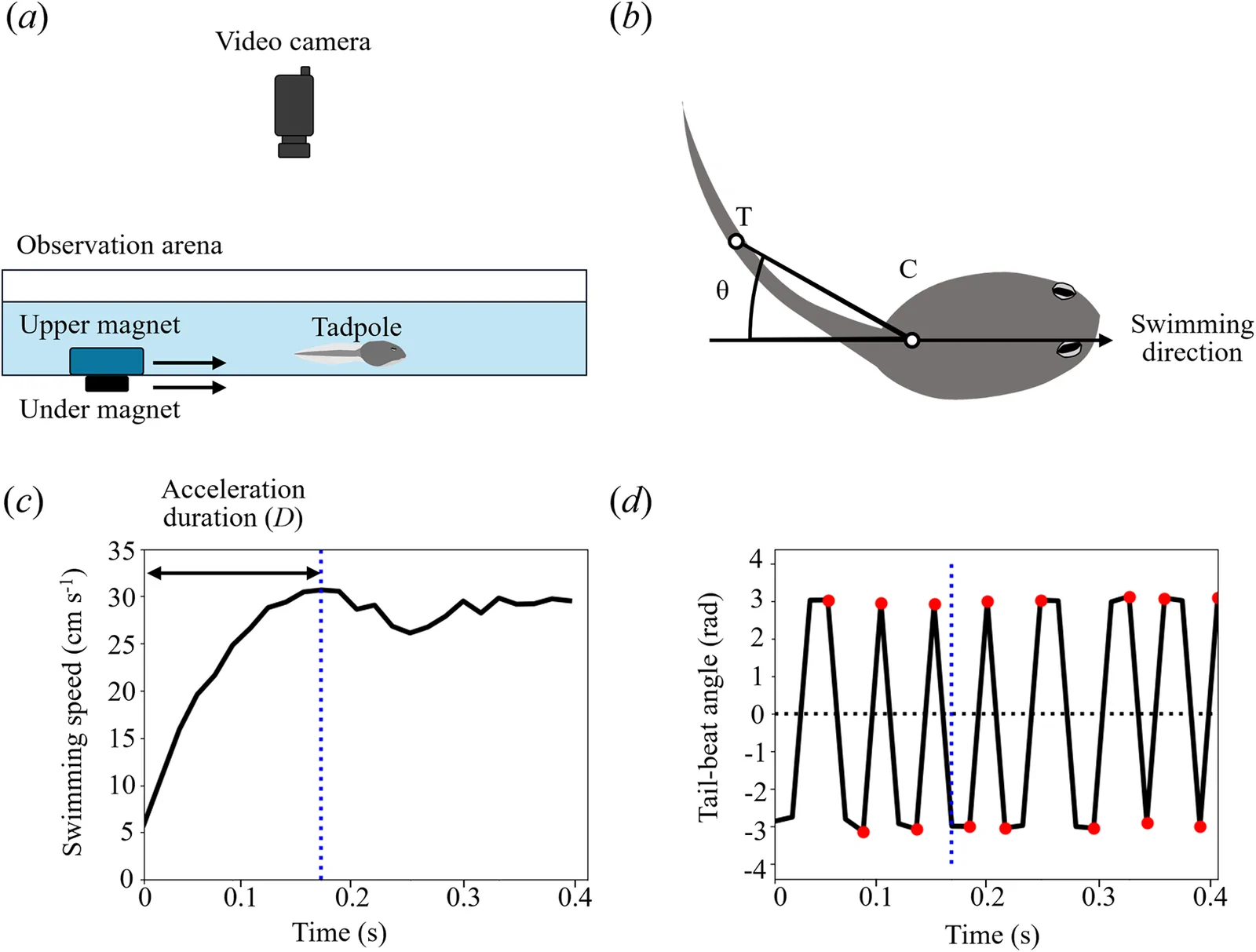
Escape experiment system and definitions of escape responses in *R. pirica* tadpoles. (*a*) Schematic diagram of escape experiment. (*b*) Definition of posture points and tail-beat angle. The body centre (C) and the tail midpoint (T) are indicated by black-outlined white circles. θ denotes the tail-beat angle relative to the swimming direction (0°). (*c*) Time-series changes in fast-start swimming speed. The blue dashed line marks the end of the acceleration duration (*D*) (see Materials and methods). (*d*) Time-series changes in tail beat. The moving direction is indicated by an angle of 0. The red point represents the peak of the tail beat. The blue dashed line indicates the end time of the acceleration duration.

To quantify the morphological traits, tadpoles used in the escape experiment were photographed from dorsal and lateral views. We digitally measured the following six morphological traits of tadpoles with ImageJ (https://imagej.nih.gov/ij/index.html, version 1.53): body length (the tip of the snout to the most posterior point of the body excluding the cloacal tailpiece), tail length, maximum body depth, maximum body width, maximum tail depth and maximum tail muscle depth (supplementary material S2).

### 2.4. Analytical framework for separating morphology and body-motion effort

In the present study, we focused on swimming speed as a component of fast-start swimming performance. As outlined in the Introduction, swimming speed reflects both body-motion effort and the effectiveness with which that effort is converted into forward speed. To isolate the latter component, we evaluated the effectiveness with which body-motion effort is converted into swimming speed (hereafter, *E_ms_*). Differences in *E_ms_* were therefore interpreted as reflecting differences in the contribution of morphology to swimming performance. To calculate body-motion effort, we quantified tail kinematics in addition to swimming speed.

### 2.5. Quantification of fast-start swimming speed and body-motion effort

Escape responses of tadpoles were extracted from videos (supplementary material S1). We excluded trials in which tadpoles collided with the tank wall before reaching maximum swimming speed, because such collisions could affect both body motion and swimming performance. We also excluded trials involving pronounced initial reorientation approaching 180°, because the large tail movement associated with initial reorientation was not periodic, preventing tail-beat frequency from being quantified in a manner comparable to that of other escape responses (described below). Only tadpoles that displayed three eligible escape responses out of five attempts were used for subsequent analysis (i.e. non-induced and dragonfly-induced phenotype: nine individuals each, salamander-induced phenotype: 10 individuals). To analyse the escape responses, we used an automated, open-source animal tracking software, TRex [36], and processed and analysed the extracted data using Python (version 3.12.0).

To quantify swimming speed, we first obtained the speed profile of each escape response by calculating instantaneous swimming speed for every video frame. For this purpose, the position of the body centre point (point C; figure 2*b*) was estimated using TRex's weighted centroid (X#wcentroid, Y#wcentroid), which is computed by thresholding pixels to extract a body blob and calculating its intensity-weighted centre, such that brighter body pixels contribute more to the estimated position. Compared with the unweighted centroid, this weighted centroid provides a more stable estimate of body-centre position in fast-moving tadpoles because it is less affected by tail flicks or small changes in body outline. Instantaneous swimming speed was calculated from the temporal displacement of point C and smoothed using a 3-frame (0.05 s) moving average to reduce noise.

We used *post-acceleration swimming speed* (*V*) as an operational measure of swimming speed because it usually corresponded to the maximum swimming speed. To determine *V* from the speed profile, we first identified the *acceleration duration* (*D*), which was defined as the period from the onset of swimming until the first frame at which the smoothed swimming speed decreased relative to the previous frame (figure *2c*). *V* was then defined as the smoothed swimming speed at the end of the acceleration duration.

Next, to examine the body-motion effort during fast-start swimming, we quantified the motion of the tail—the main thrust-producing structure in tadpoles [37]. As an index of body-motion effort during fast-start swimming, we calculated *tail-motion quantity during acceleration* (*TQ*) from two tail-kinematic variables: *tail-beat amplitude* (*A*) and *tail-beat frequency* (*f*), together with *D*. Because the mid-tail region generates the primary propulsive thrust in tadpoles [38], *A* and *f* were measured at the tail midpoint. The tail midpoint was defined as a point located approximately halfway along the tail centreline from the tail base to the tail tip and designated as the tail point (point T; figure 2*b*; for a detailed explanation, see supplementary material S3).

To quantify *A*, we first calculated a tail-angle time series during the *D*. Tail angle was defined as the angle between the movement direction, estimated from the temporal change in point C, and the line segment connecting points C and T (figure 2*b*, *d*). Peaks in the tail-angle time series were detected using the ‘find_peaks’ function of the SciPy package. Here, a peak refers to each maximum lateral displacement of the tail midpoint during a tail-beat cycle. Average *A* was then calculated as the mean amplitude across all detected peaks within the *D*.

*f* was quantified from periodic tail-beat cycles during the acceleration duration based on the timing and number of detected peaks. Specifically, *f* was calculated as

(2.1) $$f=\frac{\frac{\left(N-1\right)}{2}}{t_{last}-t_{first}},$$

where *N* is the number of tail-beat peaks within *D*, and *t_first_* and *t_last_* are the times of the first and last peaks, respectively. The term (*N* − 1) / 2 represents the number of tail-beat cycles between the first and last detected peaks.

*TQ* was then calculated as

(2.1) TQ = A×f×D ,

where *A* is the average tail-beat amplitude, and *D* is the acceleration duration. The *TQ* represents the total quantity of tail movement during the acceleration phase—that is, a simplified index of the amount of water displaced to propel the body forward.

### 2.6. Operational evaluation of motion–speed efficiency

After quantifying *V* and *TQ*, we evaluated *E_ms_* as the effectiveness with which body-motion effort, quantified as *TQ*, is converted into swimming speed, quantified as *V*. Operationally, *E_ms_* was evaluated as swimming speed adjusted for body-motion effort (see Statistical analysis).

Comparison of *E_ms_* among phenotypes assumes that *TQ* provides a comparable measure of body-motion effort across phenotypes. This assumption is satisfied only if body size (measured as body length) and swimming style are effectively equivalent among phenotypes. This is because (i) body size can influence swimming kinematics and swimming performance [39] and (ii) if individuals use different swimming styles (i.e. overall body motion; cf. [40]), tail-motion quantity no longer provides a comparable measure of body-motion effort across phenotypes. Consequently, controlling for body-motion effort cannot reliably isolate morphological effects.

To minimize potential violations of these assumptions, we selected individuals of similar body length across phenotypes. We additionally verified that swimming style was comparable among phenotypes by comparing head amplitude, head frequency and the head : tail amplitude ratio (supplementary material S4).

### 2.7. Statistical analysis

Under the null hypothesis that the mean body length of tadpoles used in the escape experiment did not differ among the three phenotypes, one-way ANOVA was conducted, and the result showed that the body length was not significantly different among phenotypes (see Results). Because the other five morphological traits (tail length, maximum body depth, maximum body width, maximum tail depth and maximum tail muscle depth) covary with body length, we adjusted for this effect by using analysis of covariance (ANCOVA) to compare these five morphological traits among the three phenotypes. The ANCOVA results showed that four morphological traits, other than tail length, significantly differed among the phenotypes. Therefore, post hoc pairwise comparisons among the three phenotypes were conducted using the Holm–Bonferroni method [41] to control the family-wise *α* at 0.05.

To compare fast-start swimming speed or tail motion among phenotypes, we employed linear mixed models (LMMs) for our analysis. In this model, the response variable was either *V*, *A*, *f*, *D* or *TQ*. Phenotype (non-induced, salamander-induced and dragonfly-induced) and body length were included as the explanatory variables. To account for the three escape responses recorded per tadpole, individual identity was included as a random effect in the models. Body length was explicitly included as a covariate to control for individual-level size effects on swimming kinematics and performance. In all LMMs, we assessed the overall phenotype effect using a Type II ANOVA, with denominator degrees of freedom estimated using the Satterthwaite approximation, and conducted post hoc pairwise comparisons with Holm–Bonferroni–adjusted *P*-values (family-wise *α* = 0.05).

To compare *E_ms_* among phenotypes, we modelled *V* as a function of *TQ* using a multiple-predictor LMM. In the LMM, *V* was the response variable, and mean-centred *TQ*, phenotype, their interaction and body length were included as explanatory variables, with individual identity as a random effect to account for repeated measurements within tadpoles. Body length was explicitly included as a covariate to control for individual-level size effects on swimming speed. Because *TQ* was mean-centred, phenotype-specific intercepts represent predicted swimming speeds at the average level of tail-motion effort.

Under this modelling framework, phenotypic differences in *E_ms_* could be detected as a phenotype main effect, a *TQ* × phenotype interaction, or both. A phenotype main effect represents differences in swimming speed among phenotypes at a common level of tail-motion effort, whereas a *TQ* × phenotype interaction represents differences among phenotypes in the increase in swimming speed achieved per unit increase in tail-motion effort. The interpretation of these model terms is illustrated conceptually in supplementary material S5. We assessed overall phenotype effect and the *TQ* × phenotype interaction using a Type III ANOVA and conducted post hoc pairwise comparisons with Holm–Bonferroni–adjusted *P*-values (family-wise *α* = 0.05).

We also examined whether variation in escape-trial conditions could have influenced the results. Stimulus speed and the distance from the tadpole to the nearest tank wall at the onset of escape were considered potential confounding factors because they could affect tail kinematics or *E_ms_*. We tested whether these variables differed among phenotypes, examined their effects on tail-beat frequency and amplitude and added both variables to the *E_ms_* model to assess whether the main conclusions were robust to their inclusion. These supplementary checks confirmed that accounting for stimulus speed and wall distance did not alter the qualitative conclusions of the main analyses (supplementary material S6).

Because induction tank identity was not recorded for each individual, we were unable to include tank as a random effect in the statistical analyses, and some degree of non-independence among individuals from the same tank cannot be ruled out. To evaluate the robustness of our conclusions, we conducted supplementary analyses accounting for potential tank-level effects; the results were qualitatively unchanged (see supplementary material S7).

## 3. Results

### 3.1. Morphological traits

The body length of the tadpoles showed no significant difference among phenotypes (ANOVA, *F*_2,25_ = 0.70, *P* = 0.51). ANCOVA revealed no significant difference in tail length among phenotypes after controlling for body length (*F*_2,24_ = 1.33, *P* = 0.285, supplementary material table S8a), whereas all other size-adjusted morphological traits differed significantly in maximum body depth (*F*_2,24_ = 84.32, *P* < 0.001), maximum body width (*F*_2,24_ = 67.42, *P* < 0.001), maximum tail depth (*F*_2,24_ = 21.59, *P* < 0.001) and maximum tail muscle depth (*F*_2,24_ = 9.15, *P* < 0.01). Post hoc comparisons revealed that the salamander-induced phenotype had a deeper and wider body, as well as a deeper tail and tail muscle compared to the non-induced phenotype (figure 3; supplementary material table S8b). The dragonfly-induced phenotype had a wider body and a deeper tail than the non-induced phenotype, but a narrower body compared to the salamander-induced phenotype (figure 3; supplementary material table S8b).

**Figure 3.**
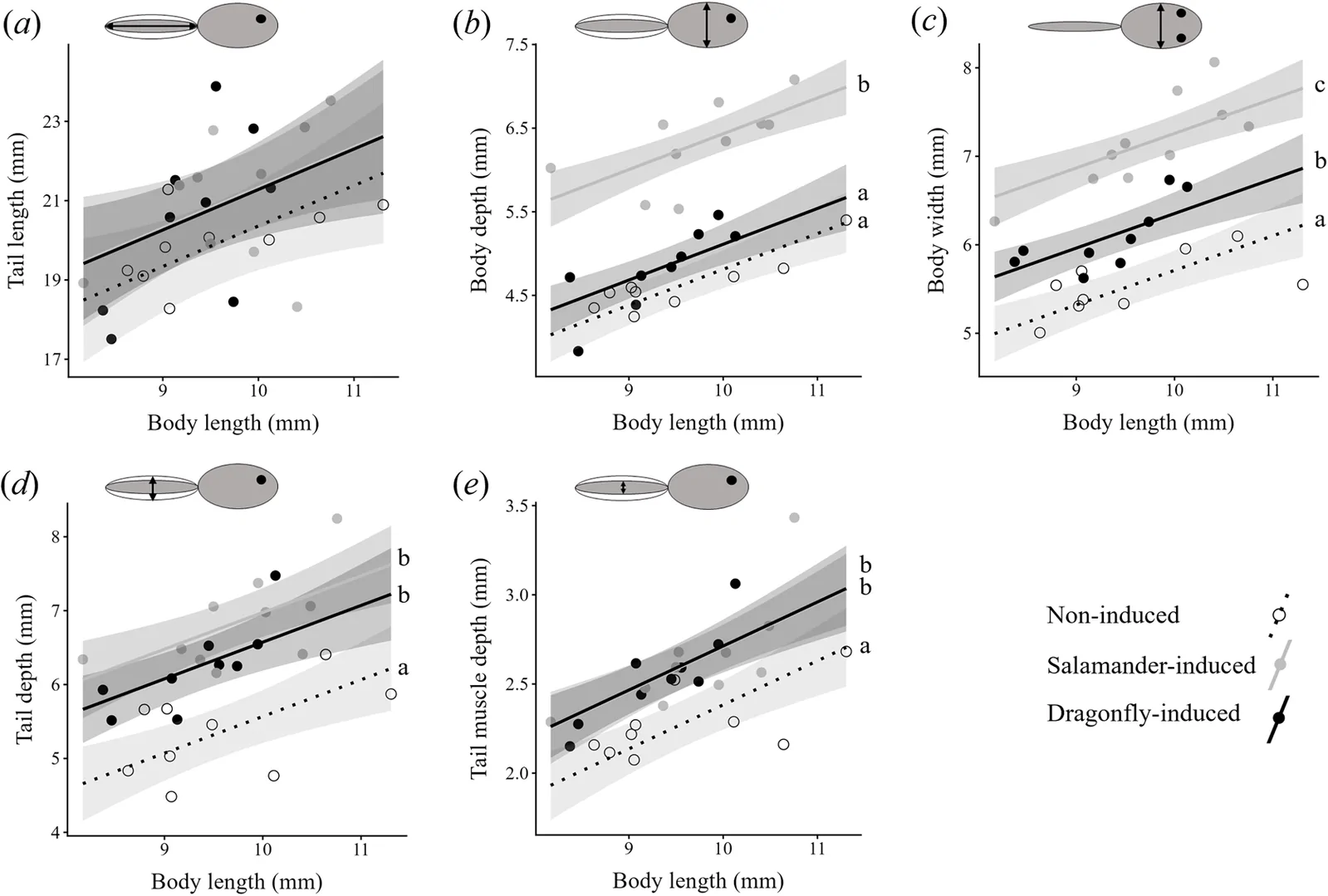
Morphological traits differed consistently among the three phenotypes. The figure shows each focal trait plotted against body length for individual tadpoles: (*a*) tail length, (*b*) body depth, (*c*) body width, (*d*) tail depth, and (*E*) tail-muscle depth. Open circles and dashed lines indicate the non-induced phenotype, grey circles and lines the salamander-induced phenotype and black circles with solid lines the dragonfly-induced phenotype. Points represent individuals and lines indicate predicted trait values for each phenotype. Shaded areas around the regression lines represent 95% confidence intervals. Phenotype effects were tested using ANCOVA with post hoc pairwise comparisons; statistically significant differences are indicated by different alphabetical letters. As the phenotype × body-length interaction was not statistically significant, regression lines are shown with a common slope across phenotypes.

### 3.2. Swimming speed

*V* differed significantly among phenotypes (Type II ANOVA, *F*_2,24_ = 13.39, *P* < 0.001, figure 4, supplemen tary material table S9). Post hoc comparisons showed that both predator-induced phenotypes exhibited higher swimming speeds than the non-induced phenotype (dragonfly-induced phenotype; *P* < 0.001, salamander-induced phenotype; *P* = 0.014). Swimming speed was also significantly higher in the dragonfly-induced phenotype than in the salamander-induced phenotype (*P* = 0.035).

**Figure 4.**
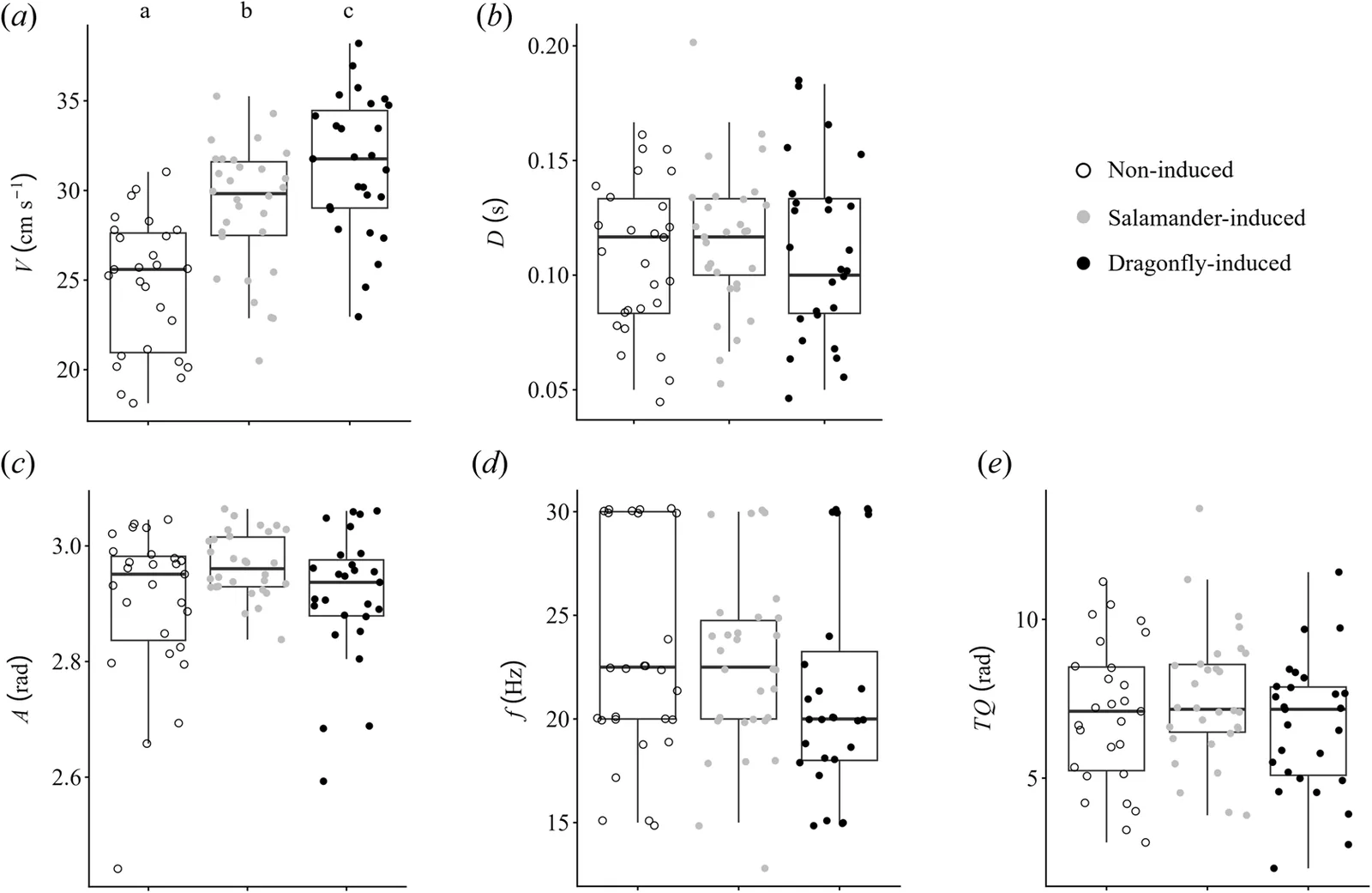
Swimming speed was highest in the dragonfly-induced phenotype, intermediate in the salamander-induced phenotype, and lowest in the non-induced phenotype. The box plots compare (*a*) post-acceleration swimming speed (*V*; cm s⁻¹), (*b*) acceleration duration (*D*; s), (*c*) average tail-beat amplitude (*A*; rad), (*d*) tail-beat frequency (*f*; Hz) and (*E*) tail-motion quantity during acceleration (*TQ*; rad) across the three phenotypes. Open circles represent the non-induced phenotype, grey circles the salamander-induced phenotype and black circles the dragonfly-induced phenotype. Each point represents a single escape response (three responses per individual). Boxes show the interquartile range (IQR) with the median indicated by a horizontal line; whiskers extend to 1.5 × IQR. Statistically significant differences among phenotypes are indicated by different alphabetical letters, based on post hoc pairwise comparisons following the linear mixed models.

### 3.3. Tail motion

There were no significant differences among phenotypes in *D* (*F*_2,24_ = 0.14, *P* = 0.873), *A* (*F*_2,24_ = 1.61, *P* = 0.220), *f* (*F*_2,24_ = 0.61, *P* = 0.551) or *TQ* (*F*_2,80_ = 0.78, *P* = 0.464) (Type II ANOVA, figure 4, supplementary material table S9).

### 3.4. Motion–speed efficiency (*E_ms_*)

The LMM examining *E_ms_* revealed a significant overall effect of phenotype, whereas the interaction between *TQ* and phenotype was not significant (Type III ANOVA, phenotype: *F*_2, 23.67_ = 16.70, *P* < 0.001; *TQ* × phenotype: *F*_2, 72.18_ = 0.72, *P* = 0.489; table 1). Post hoc comparison showed that the phenotype-specific intercept was highest in the dragonfly-induced phenotype (versus salamander-induced phenotype; *P* = 0.01, vs non-induced phenotype; *P* < 0.001), followed by the salamander-induced phenotype (versus non-induced phenotype; *P* = 0.01), and lowest in the non-induced phenotype (figure 5). This implies that *E_ms_* was highest in the dragonfly-induced phenotype, intermediate in the salamander-induced phenotype and lowest in the non-induced phenotype.

**Table 1.** Results of the LMM testing differences in *E_ms_* among phenotypes. Non-induced phenotype was used as the reference category. Asterisks indicate significant differences: * for *p* < 0.05, ** for *p* < 0.01, and *** for *p* < 0.001.

effect	estimate	std. error	*t* value	d.f.	*F* value	*P* value
**fixed effect (LMM)**						
intercept	13.93	6.76	2.06			0.050
tail-motion quantity (*TQ*)	0.32	0.28	1.14			0.260
phenotype (salamander-induced)	3.46	1.16	2.97			0.007^**^
phenotype (dragonfly-induced)	6.72	1.16	5.78			<0.001^***^
body length	0.37	0.23	1.61			0.120
*TQ* × phenotype (salamander-induced)	0.47	0.39	1.19			0.238
*TQ* × phenotype (dragonfly-induced)	0.30	0.41	0.72			0.475
**ANOVA (Type III)**						
phenotype (overall effect)				2, 23.67	16.70	<0.001^***^
*TQ* × phenotype (overall effect)				2, 72.18	0.72	0.489

**Figure 5.**
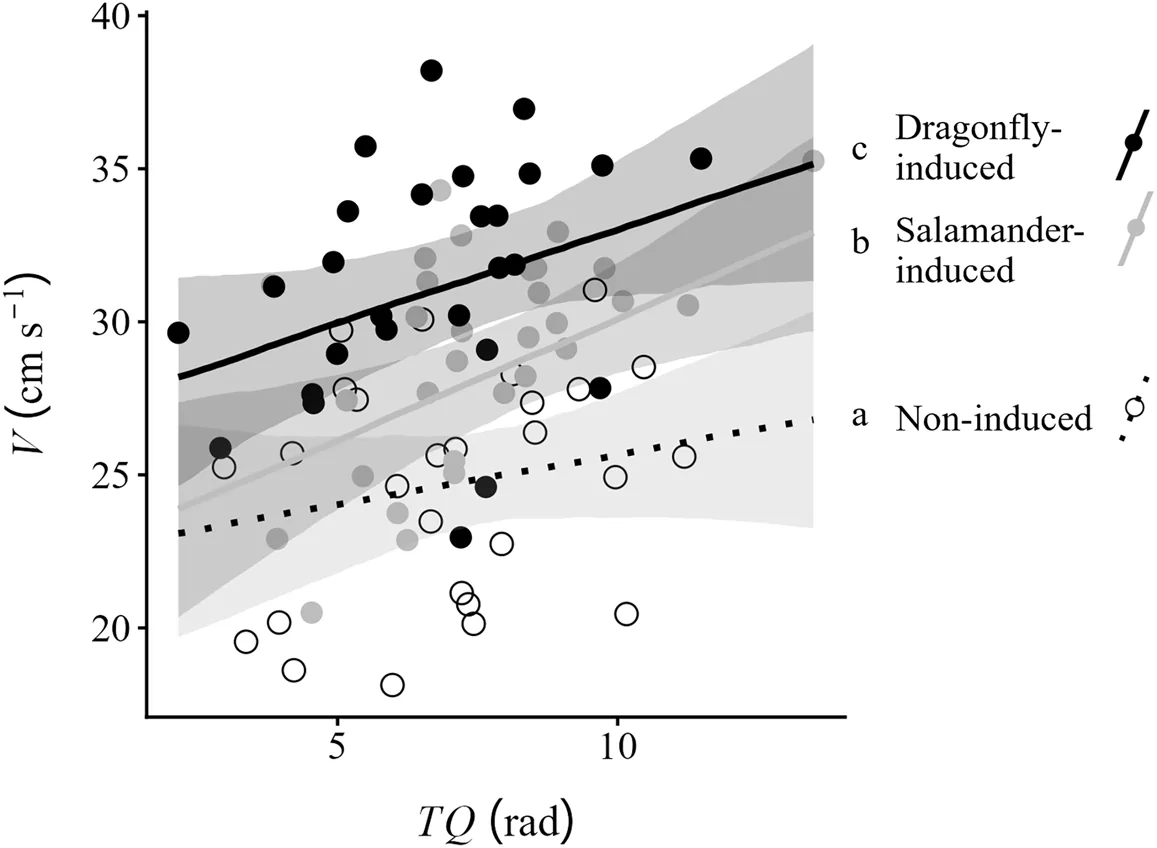
*E_ms_* differed among phenotypes. *V* is plotted against *TQ*; each point represents a single escape response (three per individual). Open circles and dashed lines indicate the non-induced phenotype, grey circles and lines the salamander-induced phenotype and black circles and solid black lines the dragonfly-induced phenotype. Lines show fixed-effects predictions from the LMM including the *TQ* × phenotype interaction, with body length fixed at its mean and random effects excluded. Shaded areas indicate 95% confidence intervals. Different letters indicate statistically significant differences among phenotypes at the mean level of *TQ*.

## 4. Discussion

Many studies have shown that predator-induced phenotypes exhibit greater fast-start swimming compared to non-induced phenotypes [21–23], yet it has remained elusive whether morphological changes themselves contribute to this enhancement. Here, in *Rana pirica* tadpoles, we demonstrate that both salamander-induced and dragonfly-induced phenotypes exhibited distinct morphological traits and achieved higher fast-start speed (*V*) compared to the non-induced phenotype, consistent with the general pattern reported in previous studies. Crucially, we also found that both predator-induced phenotypes exhibited enhanced *E_ms_* compared to non-induced phenotypes. Our results suggest that predator-induced morphological changes promote efficient propulsion, thereby enhancing fast-start swimming performance in *R. pirica* tadpoles. Notably, our findings provide the first empirical test of the broader hypothesis that plastic morphological changes, beyond the behavioural changes alone, affect swimming speed in aquatic animals [21,23–27].

Morphological traits that serve one function can constrain another, highlighting the functional trade-offs inherent in morphology [1,3]. In *R. pirica* tadpoles, we hypothesized a functional trade-off between escape and protection underlying predator-induced morphological traits, and our results supported this hypothesis. Tail size (i.e. overall tail fin depth and tail muscle depth), a key determinant of tadpole propulsion [37], was larger in both the salamander-induced and dragonfly-induced phenotypes than in the non-induced phenotype. Consistent with the prediction from previous studies—in which larger tails are expected to generate greater thrust and thereby improve the efficiency with which tail-motion effort is converted into forward speed [26,34,42]—both predator-induced phenotypes exhibited higher *E_ms_* than the non-induced phenotype in *R. pirica* tadpoles. However, although both predator-induced phenotypes possessed similarly enlarged tails, the dragonfly-induced phenotype exhibited higher *E_ms_* than the salamander-induced phenotype. The most plausible morphological mechanism underlying the difference in *E_ms_* is that the salamander-induced phenotype has a larger body surface area owing to its increased body depth and width, which likely increases hydrodynamic drag and offsets the thrust advantage conferred by its enlarged tail. Hydrodynamic drag is theoretically predicted to increase in proportion to body surface area [43], supporting this interpretation. The enlarged body of the salamander-induced phenotype has been experimentally shown to prevent swallowing predation by exceeding the gape of salamanders [29], demonstrating a clear protective function. Taken together, these findings indicate that the salamander-induced phenotype represents a morphological strategy that prioritizes protection against gape-limited predators, even at the cost of reduced efficiency in converting tail-motion effort into forward speed owing to increased drag. By contrast, the dragonfly-induced phenotype—expressed under the risk of biting-type predators—exhibits a morphology specialized for escape, achieving rapid evasion with effective propulsion. Thus, the two predator-induced phenotypes exemplify distinct optimizations of functional trade-offs shaped by the contrasting predation modes they face.

We showed that tadpoles with deeper tails exhibited higher fast-start escape speeds in response to stimuli applied from the lateral or posterior direction. Such higher escape speeds may increase the probability of evading short-range strikes by ambush predators such as dragonflies and salamander larvae [44]. Fast-start escape responses may involve both an initial reorientation away from the stimulus and subsequent forward propulsion to leave the predator's strike range [28]. Rapid initial reorientation may allow prey to escape along a shorter path, whereas faster forward propulsion may allow them to leave the strike range more quickly [44–45]. The relative importance of these components for predator evasion may therefore depend on the conditions of the predator–prey encounter, including the direction and distance of the predator's approach [45]. In the present study, stimuli were applied from the lateral or posterior direction, and responses involving pronounced initial reorientation were excluded from the analyses. The responses examined here therefore primarily represented escapes involving relatively limited initial reorientation. Nevertheless, some degree of reorientation may also have occurred in the responses included in the analyses. Because the forces generated during initial reorientation may also contribute to translational acceleration, we cannot determine whether deeper tails increased escape speed by improving one or both of these components. Future studies should quantify turning and forward propulsion separately to determine how deeper tails affect the different locomotor components of fast-start escape performance.

Our results demonstrate that both predator-induced phenotypes achieved greater swimming speed than the non-induced phenotype at a given level of body-motion effort. While a deeper tail fin may enhance thrust production during tail movement, moving a larger tail structure can itself require greater muscular effort (i.e. increased inertial loading during tail acceleration) and exposes a larger surface area to water-borne forces (i.e. greater viscous drag) compared to shallower tails. Likewise, the swollen body likely requires stronger propulsive force to overcome increased frontal resistance (i.e. greater pressure drag) compared to more streamlined bodies. Morphologies that increase hydrodynamic resistance are known to elevate the energetic cost of locomotion [46], and both the deeper tail and swollen body may therefore increase the energetic expenditure required to achieve a given swimming performance. Elevated locomotor energy expenditure could impose fitness costs by diverting resources away from other functions, such as growth and development [47–49]. Such energetic costs likely constitute one component of the fitness trade-offs associated with predator-induced morphologies, helping to explain why these defensive traits are expressed only under predation risk rather than constitutively. In general, predator-induced morphologies are assumed to be inducible because they carry fitness costs, but empirically demonstrating these costs has proven extremely challenging [50]. Many studies have inferred the presence of such costs by showing that individuals raised under predation risk exhibit reduced growth or survival compared to those raised without risk (e.g. [51–53]). However, under predation risk, individuals typically alter not only their morphology but also their behaviour and life-history traits [50,54–55]. Consequently, it is difficult to determine whether reduced growth or survival arises specifically from the morphological traits themselves or from other correlated responses. For this reason, mechanistically testing whether predator-induced morphologies incur energetic costs is crucial for understanding the evolution of inducible defensive traits. Although we did not directly measure energetic expenditure, our results provide indirect evidence that such costs may arise. Specifically, the salamander-induced phenotype exhibited lower *E_ms_* than the dragonfly-induced phenotype despite possessing a similarly enlarged tail, indicating that greater body enlargement reduces the effectiveness with which locomotor effort is converted into forward speed. If lower *E_ms_* translates into greater energetic expenditure for achieving a given swimming performance, then the protective morphology may carry an energetic cost that contributes to the fitness trade-offs maintaining inducible defences. Future studies should directly test this possibility by comparing the energetic expenditure required to achieve a given swimming performance (e.g. oxygen consumption per unit swimming speed [56]) among predator-induced and non-induced phenotypes. Quantifying differences in locomotor energy requirements would provide a basis for evaluating whether energetic trade-offs translate into measurable fitness costs.

Our results show that morphological variation generated by predator-induced plasticity in *R. pirica* tadpoles involves a trade-off between swimming performance and protection. Similar swimming-related trade-offs may also be involved in predator-induced morphological variation in other species, as well as in morphological variation induced plastically by environmental factors other than predation. For example, like the salamander-induced phenotype of *R. pirica* tadpoles, several predator-induced phenotypes exhibit prominent defensive structures, such as swollen bodies and spines in dragonfly naiads [57–58] and zooplankton [59–63]. These traits may enhance protective functions by making prey more difficult to swallow for gape-limited predators [64–65] or by reducing predator handling efficiency, even if they reduce the effectiveness with which body motion is translated into forward propulsion. Conversely, environmental conditions that increase the demand for swimming, such as stronger flow or intensified competition [33,66–69], may induce morphological traits that improve locomotor performance, but such changes may also compromise other functions that are advantageous under different environmental conditions. Accumulating evidence from diverse cases of plastic morphological variation will help clarify how widely swimming-related trade-offs are involved in environmentally induced morphological variation.

The diversity of individual body forms observed in aquatic animals is generated across multiple biological levels, including both evolutionary divergence among species and morphological variation within species. Trade-offs between swimming performance and other functions have been proposed as one mechanism shaping morphological divergence among aquatic species, and our results extend this perspective to plastic variation in morphology within species. Our findings therefore suggest that swimming-related trade-offs are an important functional basis underlying the diverse body forms of mobile aquatic animals across biological levels.

## Supplementary Material



## Data Availability

Data and code supporting this article have been deposited in Figshare and are publicly available at [70]. Supplementary material is available online [71].
