# Supplementary material for: Predator-induced morphological traits determine escape performance in a larval amphibian species
Source: R Soc Open Sci. 2026 Oct 14;13(10):260411. doi: 10.1098/rsos.260411 (PMC13682424; doi:10.1098/rsos.260411)
Supplement: Supplementary file 1 [file rsos.260411.zip › rsos.260411/rsos260411_si_001.docx]

**Predator-induced morphological traits determine escape performance in a larval amphibian species**

**Supplementary material**

**Supplementary material S1.** A Video of the escape responses of *R. pirica* tadpole.

As an example, we provide the video of the first escape response of individual ID 21 (see the corresponding row in the CSV data file) from the dragonfly-induced phenotype. The red dot indicates the body centroid, the green line shows the extracted midline, and the green dot marks the detected tail tip (located approximately at the mid-point of the tail; see Supplementary Material S3). The yellow line represents the swimming direction.

DOI: 10.6084/m9.figshare.30866090

**Supplementary material S2.** Visual clarification of morphological trait definitions and measurement locations.

**Figure S2.** Representative dorsal and lateral photographs of tadpoles (non-induced phenotype) showing the morphological measurements used in this study. Red arrows show (a) body length (the tip of snout to the most posterior point of the body excluding the cloacal tailpiece), (b) tail length, (c) maximum body depth, (d) maximum body width, (e) maximum tail depth, and (f) maximum tail muscle depth.

**Supplementary material S3.** Accuracy of tail-midpoint detection from video.

Because TRex relies on a background-subtraction–based detection algorithm [36], the faint distal portion of the tadpole tail was often not reliably detected. We therefore defined the most distal reliably detectable point as the tail point (point T). If the detected tail-midpoint position differed among phenotypes, subsequent comparisons of tail-beat motion could be biased; therefore, we evaluated whether these detection positions varied across phenotypes.

**Method**

For each escape response, we calculated the mean value of the TRex parameter “midline length” (the distance from the head tip to the distal detectable tail point) during the acceleration duration. By subtracting body length (the tip of snout to the most posterior point of the body excluding the cloacal tailpiece) from this value, we obtained the detectable tail portion only. This detectable portion was then divided by the anatomical tail length to yield the *detected tail-extent ratio*—that is, the percentage of tail length from the base that was reliably detected.

To compare the detected tail-extent ratio among phenotypes, we fitted linear mixed-effects models (LMMs) with phenotype (non-induced, salamander-induced, and dragonfly-induced) as a fixed effect and individual identity as a random effect to account for the three escape responses per tadpole.

**Result**

Validation analyses showed that tail point corresponded, on average, to approximately the midpoint of the tail (55 ± 8% of tail length from the base; Figure S3). There were no significant differences among phenotypes in the detected tail-extent ratio. Specifically, detected tail-extent ratio was 56 ± 7% in the non-induced phenotype, 53 ± 8% in the salamander-induced phenotype, and 56 ± 9% in the dragonfly-induced phenotype. Thus, TRex consistently detected the tail point at roughly the mid-tail region across all phenotypes.

**Figure S3.** The box plot compares the detected tail-extent ratio among phenotypes. Each point represents an individual escape response (three responses per tadpole). Boxes show the interquartile range (IQR) with the median indicated by a horizontal line, and whiskers extend to 1.5 × IQR.

**Supplementary material S4.** Comparison of swimming modes of *R. pirica* tadpoles among phenotypes.

For comparing swimming style among phenotypes, we quantified the head motion and the ratio of head:tail amplitude, which was often used to examine swimming style of fish (referred to as [40]).

**Method**

The procedure for quantifying head-beat kinematics followed the same general approach as that used for tail-beat analysis in the main text. The anterior tip of the body centerline was identified and designated as the head point (Point H; Figure S4a). Head-beat amplitude was defined as the angle between the movement direction (i.e. the vector derived from the time-series change in Point C) and the line segment connecting Points C and H. Peaks of head beating during the acceleration phase were detected using the find_peaks function in the SciPy package, where each peak corresponds to the maximum lateral displacement of the head within a head-beat cycle. The head:tail amplitude ratio was calculated as the average head-beat amplitude divided by the average tail-beat amplitude.

To compare swimming style among phenotypes, we employed linear mixed models (LMMs) for our analysis. In this model, the response variable was either average head-beat amplitude, head-beat frequency, or head:tail amplitude. Phenotype (non-induced, salamander-induced, and dragonfly-induced) was used as the explanatory variable. To account for the three escape responses recorded per tadpole, individual identity was included as a random effect in the models. Type II ANOVA was performed to assess the overall effect of the categorical variable phenotype on the response variable.

**Result**

There were no significant differences among phenotypes in average head-beat amplitude (*F*_2,25_ = 0.05, *P* = 0.956), head-beat frequency (*F*_2,25_ = 0.46, *P* = 0.639), or head:tail amplitude (*F*_2,25_ = 0.06, *P* = 0.942) (Type II ANOVA, Figure S4b).

**Figure S4a.**The definition of posture points and angle of *R. pirica* tadpole motion. Red point of H is the head point along the centerline, red point of C is the center point of tadpoles, and θ is the head-beat angle against the moving direction of tadpoles.

**Figure S4b.** The box plot compares swimming style among phenotypes. Each point represents an individual escape response (three responses per tadpole individual). Each box represents the interquartile range (IQR) with a horizontal line at the median. Whiskers extend to 1.5 times the IQR. Panels show (a) average head-beat amplitude, (b) head-beat frequency, and (c) head:tail amplitude.

**Supplementary material S5.** Rationale for evaluating motion–speed efficiency using swimming speed adjusted for tail-motion effort

As described in the main text, we evaluated motion–speed efficiency (*𝐸_ms_*) operationally as swimming speed adjusted for tail-motion effort. Phenotypic differences in motion–speed efficiency can be interpreted either from differences in the slope of the *𝑉–𝑇𝑄* relationship or from differences in predicted *𝑉* at a common level of *𝑇𝑄*, depending on the structure of the *𝑉–𝑇𝑄* relationship. If *𝑉* increases approximately linearly with *𝑇𝑄*, differences in slope provide a straightforward interpretation: a steeper slope indicates a greater increase in swimming speed per unit increase in tail-motion effort (Figure S5a). In this case, phenotypic differences in slope can be interpreted as differences in the effectiveness with which tail-motion effort is converted into swimming speed.

However, the *𝑉–𝑇𝑄* relationship may not increase linearly across the full range of tail-motion effort. Instead, *𝑉* may rise steeply at low *𝑇𝑄* values and then gradually approach saturation as *𝑇𝑄* increases (Figure S5b). Such a saturating pattern may arise because greater tail-motion effort incurs increasing losses in converting motion into forward speed, for example due to water drag [70]. Under this pattern, an increase in *𝑇𝑄* can produce a relatively large increase in *𝑉* when *𝑇𝑄* is low, but only a small increase in *𝑉* when *𝑇𝑄* is already high. Thus, the swimming speed gained from an additional unit of tail-motion effort depends on where along the *𝑇𝑄* range the comparison is made. Under such conditions, comparing predicted swimming speed at a common level of *𝑇𝑄* provides a clearer way to evaluate whether phenotypes differ in the effectiveness with which tail-motion effort is converted into swimming speed.

In the present data, the observed *𝑉–𝑇𝑄* relationship showed a tendency consistent with this saturating pattern, rather than a simple proportional relationship over a broad range. For the statistical comparison among phenotypes, however, we used a linear approximation of the *𝑉*–*𝑇𝑄* relationship within the observed range of *𝑇𝑄*. Under this approximation, the LMM detected a significant phenotype main effect but no significant *𝑇𝑄* × phenotype interaction. We therefore evaluated motion–speed efficiency primarily as phenotypic differences in predicted swimming speed at a common level of tail-motion effort. Because *𝑇𝑄* was centered before model fitting, this common level corresponded to the average observed *𝑇𝑄*; thus, phenotype-specific intercepts represent predicted swimming speeds at the average level of tail-motion effort.

**Figure S5.** Conceptual rationale for evaluating motion–speed efficiency.

(a) Conceptual example of a proportional relationship between post-acceleration swimming speed (*𝑉*) and tail-motion quantity during acceleration (*𝑇𝑄*). If *𝑉* increases approximately in proportion to *𝑇𝑄*, phenotypic differences in motion–speed efficiency can be represented by differences in the slopes of the *𝑉–𝑇𝑄* relationship. The red shaded area indicates the assumed *𝑇𝑄* range corresponding to the observed data, and the red lines illustrate slope differences within this range.

(b) Conceptual overview of a saturating *𝑉–𝑇𝑄* relationship. In this case, phenotypic differences within the observed *𝑇𝑄* range were evaluated using a linear approximation. The red shaded area indicates the approximate *𝑇𝑄* range observed in the present study, and the red lines illustrate this approximation. Vertical differences among these lines represent differences in swimming speed at a common level of *𝑇𝑄*, which were interpreted as relative differences in motion–speed efficiency.

**Supplementary material S6.** Effects of stimulus speed and wall proximity on escape-trial results.

To ensure that differences in escape responses among phenotypes were not attributable to variation in the stimulation procedure, we performed supplementary analyses examining two potential confounding factors: stimulus speed and tadpole position at the time of stimulation (wall proximity). First, we tested whether these factors differed among phenotypes. Second, because variation in stimulus speed or wall proximity could influence escape movements, we examined their effects on tail-beat frequency and tail-beat amplitude. Finally, we added both factors to the motion–speed efficiency model to determine whether they affected post-acceleration swimming speed after accounting for tail-motion quantity or altered the main conclusions regarding phenotypic differences.

**(a) Did stimulus speed differ among phenotypes?**

We quantified the mean speed of the magnetic stimulus during each escape trial and tested whether it differed among phenotypes. Mean stimulus speed did not differ among phenotypes (LMM: *F*_2,75_ = 0.83, *P* = 0.440), indicating that stimulus presentation was comparable across treatments.

**(b) Did wall proximity at the onset of escape differ among phenotypes?**

Wall proximity could potentially influence tail motion or hydrodynamic conditions during escape. As described in the Methods, trials in which tadpoles contacted the wall before reaching maximum escape speed were excluded from the main analyses. No tadpoles contacted the magnetic stimulus during the analyzed trials, and all analyzed escape responses were directed away from the approaching stimulus. Nevertheless, we performed additional analyses to evaluate whether wall proximity could have influenced our conclusions.

The distance to the nearest wall at the onset of escape averaged 19.3 ± 15.1 mm (mean ± SD; range = 7.4–91.6 mm) and did not differ among phenotypes (LMM: *F*_2,81_ = 0.018, *P* = 0.982).

**(c) Did stimulus speed and wall proximity influence swimming kinematics or motion–speed efficiency?**

We next examined whether stimulus speed or wall distance influenced swimming kinematics. Neither stimulus speed nor wall distance had a significant effect on tail-beat frequency (stimulus speed: *P* = 0.111; wall distance: *P* = 0.799) or tail-beat amplitude (stimulus speed: *P* = 0.890; wall distance: *P* = 0.441).

We then added both stimulus speed and wall distance as explanatory variables to the linear mixed model used to evaluate motion–speed efficiency. In this model, post-acceleration swimming speed was modelled as a function of tail-motion quantity, phenotype, their interaction, stimulus speed, wall distance, and body size, with individual identity included as a random effect. Neither stimulus speed (*P* = 0.265) nor wall distance (*P* = 0.960) had a significant effect on post-acceleration swimming speed after accounting for the other explanatory variables (Table S6). In contrast, the phenotype effect remained significant and produced the same qualitative pattern as in the main analysis. Thus, variation in stimulus speed and wall proximity did not explain the observed phenotypic differences in swimming kinematics or motion–speed efficiency.

**Table S6.** Results of the LMM testing differences in motion–speed efficiency (*𝐸_ms_*) among phenotypes while accounting for stimulus speed and wall distance. Non-induced phenotype was used as the reference category. Asterisks indicate significant differences: * for P < 0.05, ** for P < 0.01, and *** for P < 0.001.

| Effect | Estimate | Std. Error | t value | Df | F value | P value |
| --- | --- | --- | --- | --- | --- | --- |
| **Fixed Effect (LMM)** |  |  |  |  |  |  |
| Intercept | 15.64 | 6.91 | 2.26 |  |  | 0.033^*^ |
| Tail-motion quantity (*𝑇𝑄*) | 0.16 | 0.29 | 0.55 |  |  | 0.585 |
| Phenotype (salamander-induced) | 3.45 | 1.20 | 3.04 |  |  | 0.005^**^ |
| Phenotype (dragonfly-induced) | 6.65 | 1.20 | 3.04 |  |  | <0.001^***^ |
| **Stimulus speed** | -0.85 | 0.08 | -1.13 |  |  | 0.265 |
| **Wall distance** | 1.36E-03 | 0.03 | 0.05 |  |  | 0.960 |
| Body length | 0.332 | 0.23 | 1.45 |  |  | 0.161 |
| *𝑇𝑄* × Phenotype (salamander-induced) | 0.47 | 0.39 | 1.19 |  |  | 0.238 |
| *𝑇𝑄* × Phenotype (dragonfly-induced) | 0.30 | 0.41 | 0.72 |  |  | 0.475 |
| **ANOVA (Type III)** |  |  |  |  |  |  |
| Phenotype (overall effect) |  |  |  | 2, 23.67 | 16.70 | <0.001^***^ |
| *𝑇𝑄* × Phenotype (overall effect) |  |  |  | 2, 72.18 | 0.72 | 0.489 |

**Supplementary material S7.** Sensitivity analyses for potential tank-level non-independence.

Because tank identity was not recorded for individuals used in the escape trials, we could not include tank as a random effect. To evaluate whether potential within-tank non-independence could influence our main conclusion regarding motion–speed efficiency, we conducted two complementary sensitivity analyses.

First, we refitted the main motion-speed efficiency model using individual means, such that each individual contributed a single data point (i.e., repeated escape trials were averaged per individual). In this analysis, mean post-acceleration swimming speed was modeled as a function of mean tail-motion quantity during acceleration, phenotype, and body size. Under this framework, phenotypic differences in intercept represent differences in motion–speed efficiency as in the main analysis.

Post-hoc comparisons with Holm adjustment confirmed the same rank order in motion–speed efficiency as in the main analysis. The dragonfly-induced phenotype exhibited the highest intercept, differing significantly from both the salamander-induced phenotype (*P* = 0.015) and the non-induced phenotype (*P* < 0.0001). The salamander-induced phenotype also differed significantly from the non-induced phenotype (*P* = 0.015).

Second, we implemented a conservative stratified cluster bootstrap to account for possible within-tank dependence. Within each phenotype, individuals were randomly grouped into pseudo-tanks of size k=4, corresponding to the maximum number of individuals selected from a single induction tank. Resampling was then conducted at the pseudo-tank level with replacement, and the individual-mean model was refitted for each bootstrap replicate.

Across bootstrap replicates, both induced phenotypes consistently exhibited higher intercepts than the non-induced phenotype. The dragonfly-induced effect relative to the non-induced phenotype was positive in 100% of replicates (95% bootstrap interval: 4.15–9.93), and the salamander-induced effect relative to the non-induced phenotype was positive in 99.7% of replicates (95% interval: 1.36–5.84). In addition, the difference between induced phenotypes (dragonfly − salamander) was positive in 99.1% of replicates (95% interval: 1.21–5.70).

Together, these analyses indicate that the inference that predator-induced phenotypes exhibit higher motion–speed efficiency than the non-induced phenotype, and that the dragonfly-induced phenotype exhibits higher motion–speed efficiency than the salamander-induced phenotype, is robust to conservative assumptions of strong within-tank clustering.

**Supplementary material S8.** Results of statistical analysis comparing morphological traits among phenotypes.

**Table S8a.** Results of ANCOVA comparing morphological traits among phenotypes. Asterisks indicate significant differences: * for P < 0.05, ** for P < 0.01, and *** for P < 0.001.

| Morphological traits | Fixed Effects Variables | Df | Sum Sq | Mean Sq | F value | P value |
| --- | --- | --- | --- | --- | --- | --- |
| Tail length | Phenotype | 2 | 6.25 | 3.13 | 1.33 | 0.285 |
|  | Body length (covariate) | 1 | 15.6 | 15.6 | 6.61 | 0.017^*^ |
|  | Residuals | 24 | 56.62 | 2.36 | NA | NA |
|  |  |  |  |  |  |  |
| Body width | Phenotype | 2 | 12.89 | 6.44 | 67.42 | <0.001^***^ |
|  | Body length (covariate) | 1 | 2.29 | 2.29 | 23.92 | <0.001^***^ |
|  | Residuals | 24 | 2.29 | 0.1 | NA | NA |
|  |  |  |  |  |  |  |
| Body depth | Phenotype | 2 | 16.55 | 8.28 | 84.32 | <0.001^***^ |
|  | Body length (covariate) | 1 | 2.74 | 2.74 | 27.94 | <0.001^***^ |
|  | Residuals | 24 | 2.36 | 0.1 | NA | NA |
|  |  |  |  |  |  |  |
| Tail depth | Phenotype | 2 | 10.58 | 5.29 | 21.6 | <0.001^***^ |
|  | Body length (covariate) | 1 | 3.7 | 3.7 | 15.08 | <0.001^***^ |
|  | Residuals | 24 | 5.88 | 0.25 | NA | NA |
|  |  |  |  |  |  |  |
| Tail muscle depth | Phenotype | 2 | 0.66 | 0.33 | 9.15 | <0.01^**^ |
|  | Body length (covariate) | 1 | 0.91 | 0.91 | 25.1 | <0.001^***^ |
|  | Residuals | 24 | 0.87 | 0.04 | NA | NA |

**Table S8b.** Results of post hoc pair-wise comparisons of morphological traits among the three Phenotypes. The non-induced phenotype is referred to as "Non," the salamander-induced phenotype as "Salamander," and the dragonfly-induced phenotype as "Dragonfly." Asterisks indicate significant differences: * for P < 0.05, ** for P < 0.01, and *** for P < 0.001.

| Morphological traits | Comparison | P value |
| --- | --- | --- |
| Body width | Non vs Salamander | <0.001^***^ |
|  | Non vs Dragonfly | <0.01^**^ |
|  | Salamander vs Dragonfly | <0.001^***^ |
|  |  |  |
| Body depth | Non vs Salamander | <0.001^***^ |
|  | Non vs Dragonfly | 0.056 |
|  | Salamander vs Dragonfly | <0.001^***^ |
|  |  |  |
| Tail depth | Non vs Salamander | <0.001^***^ |
|  | Non vs Dragonfly | <0.001^***^ |
|  | Salamander vs Dragonfly | 0.098 |
|  |  |  |
| Tail muscle depth | Non vs Salamander | <0.01^**^ |
|  | Non vs Dragonfly | <0.01^**^ |
|  | Salamander vs Dragonfly | 0.94 |

**Supplementary material S9.** Results of statistical analysis comparing swimming performance and kinematics among phenotypes.

**Table S9.** Results of LMMs comparing the post-acceleration swimming speed (*V*), acceleration duration (*D*), average tail-beat amplitude (*A*), tail-beat frequency (*𝑓*), and tail motion quantity during acceleration (*𝑇𝑄*) among phenotypes (Non-induced, Salamander-induced, and Dragonfly-induced). Non-induced phenotype was used as the reference category. Asterisks indicate significant differences: * for P < 0.05, ** for P < 0.01, and *** for P < 0.001.

| Response variable | Explanatory variable | Estimate | Std. Error | t value | Df | F value | P value |
| --- | --- | --- | --- | --- | --- | --- | --- |
| *V* | **Fixed Effect (LMM)** | |  |  |  |  |  |
|  | Intercept | 10.62 | 7.22 | 1.47 |  |  | 0.154 |
|  | Phenotype (salamander-induced) | 3.69 | 1.25 | 2.95 |  |  | 0.007^**^ |
|  | Phenotype (dragonfly-induced) | 6.43 | 1.25 | 5.17 |  |  | <0.001^***^ |
|  | Body length | 0.48 | 0.24 | 1.97 |  |  | 0.061 |
|  | **ANOVA (Type II)** | |  |  |  |  |  |
|  | Phenotype (overall effect) | |  |  | 2, 24 | 13.39 | <0.001^***^ |
|  |  |  |  |  |  |  |  |
| *D* | **Fixed Effect (LMM)** | |  |  |  |  |  |
|  | Intercept | 0.07 | 0.06 | 1.14 |  |  | 0.265 |
|  | Phenotype (salamander-induced) | 5.22E-03 | 0.01 | 0.52 |  |  | 0.611 |
|  | Phenotype (dragonfly-induced) | 1.90E-03 | 0.01 | 0.19 |  |  | 0.852 |
|  | Body length | 1.39E-03 | 1.96E-03 | 0.71 |  |  | 0.487 |
|  | **ANOVA (Type II)** | |  |  |  |  |  |
|  | Phenotype (overall effect) | |  |  | 2, 24 | 0.14 | 0.873 |
|  |  |  |  |  |  |  |  |
| *A* | **Fixed Effect (LMM)** | |  |  |  |  |  |
|  | Intercept | 2.64 | 0.19 | 14.10 |  |  | <0.001^***^ |
|  | Phenotype (salamander-induced) | 0.05 | 0.03 | 1.64 |  |  | 0.114 |
|  | Phenotype (dragonfly-induced) | 5.32E-03 | 0.03 | 0.21 |  |  | 0.834 |
|  | Body length | 8.97E-03 | 6.23E-03 | 1.43 |  |  | 0.167 |
|  | **ANOVA (Type II)** | |  |  |  |  |  |
|  | Phenotype (overall effect) | |  |  | 2, 24 | 1.61 | 0.220 |
|  |  |  |  |  |  |  |  |
| *𝑓* | **Fixed Effect (LMM)** | |  |  |  |  |  |
|  | Intercept | 16.16 | 8.03 | 2.01 |  |  | 0.055 |
|  | Phenotype (salamander-induced) | -0.63 | 1.39 | -0.45 |  |  | 0.655 |
|  | Phenotype (dragonfly-induced) | -1.52 | 1.39 | -1.10 |  |  | 0.283 |
|  | Body length | 0.23 | 0.27 | 0.84 |  |  | 0.408 |
|  | **ANOVA (Type II)** | |  |  |  |  |  |
|  | Phenotype (overall effect) | |  |  | 2, 25 | 0.6 | 0.559 |
|  |  |  |  |  |  |  |  |
| *𝑇𝑄* | **Fixed Effect (LMM)** | |  |  |  |  |  |
|  | Intercept | 1.65 | 3.39 | 0.49 |  |  | 0.628 |
|  | Phenotype (salamander-induced) | 0.29 | 0.59 | 0.49 |  |  | 0.628 |
|  | Phenotype (dragonfly-induced) | -0.43 | 0.59 | -0.73 |  |  | 0.467 |
|  | Body length | 0.18 | 0.11 | 1.59 |  |  | 0.115 |
|  | **ANOVA (Type II)** | |  |  |  |  |  |
|  | Phenotype (overall effect) | |  |  | 2, 80 | 0.78 | 0.464 |
